# Comparative cytological and transcriptome analyses of *ny2* mutant delayed degeneration of tapetal cells and promotes abnormal microspore development in neo-tetraploid rice

**DOI:** 10.3389/fpls.2023.1229870

**Published:** 2023-07-17

**Authors:** Nabieu Kamara, Yamin Jiao, Weicong Huang, Lichong Cao, Lianjun Zhu, Chongchong Zhao, Xu Huang, Fimanekeni Ndaitavela Shivute, Xiangdong Liu, Jinwen Wu, Muhammad Qasim Shahid

**Affiliations:** ^1^ State Key Laboratory for Conservation and Utilization of Subtropical Agro-Bioresources, South China Agricultural University, Guangzhou, China; ^2^ Guangdong Laboratory for Lingnan Modern Agriculture, Guangzhou, China; ^3^ Guangdong Provincial Key Laboratory of Plant Molecular Breeding, South China Agricultural University, Guangzhou, China; ^4^ College of Agriculture, South China Agricultural University, Guangzhou, China; ^5^ Crop Improvement Programme, Rokupr Agricultural Research Center, Rokupr - Sierra Leone Agricultural Research Institute (SLARI), Freetown, Sierra Leone; ^6^ Multi-disciplinary Research Services, University of Namibia, Windhoek, Namibia

**Keywords:** rice, RNA-Seq, pollen sterility, carbohydrate metabolism, tapetum

## Abstract

We aimed to investigate the genetic defects related to pollen development and infertility in *NY2*, a novel tetraploid rice germplasm known as Neo-tetraploid rice. This rice variety was created through the crossbreeding and selective breeding of various autotetraploid rice lines and has previously shown high fertility. Our previous research has revealed that the *NY2* gene, encoding a eukaryotic translation initiation factor 3 subunit E, regulates pollen fertility. However, the underlying mechanism behind this fertility is yet to be understood. To shed light on this matter, we performed a combined cytological and transcriptome analysis of the *NY2* gene. Cytological analysis indicated that *ny2* underwent abnormal tapetal cells, microspore, and middle layer development, which led to pollen abortion and ultimately to male sterility. Genetic analysis revealed that the F_1_ plants showed normal fertility and an obvious advantage for seed setting compared to *ny2*. Global gene expression analysis in *ny2* revealed a total of 7545 genes were detected at the meiosis stage, and 3925 and 3620 displayed upregulation and downregulation, respectively. The genes were significantly enriched for the gene ontology (GO) term “carbohydrate metabolic process. Moreover, 9 genes related to tapetum or pollen fertility showed down-regulation, such as *OsABCG26* (ATP Binding Cassette G26), *TMS9-1* (Thermosensitive Male Sterility), *EAT1* (Programmed cell death regulatory), *KIN14M* (Kinesin Motor), *OsMT1a* (Metallothionein), and *OsSTRL2* (Atypical strictosidine synthase), which were validated by qRT-PCR. Further analyses of DEGs identified nine down-regulated transcription factor genes related to pollen development. *NY2* is an important regulator of the development of tapetum and microspore. The regulatory gene network described in this study may offer important understandings into the molecular processes that underlie fertility control in tetraploid rice.

## Introduction

Autotetraploid, as a new rice resource, has selective advantages over diploid rice, including high stable bulk density, high yield, resistance, nutrition, and larger and heavier grain size (Wang et al., 2009; Huang et al., 2009; Shahid et al., 2011; Koide et al., 2020; Chen et al., 2021; Gan et al., 2021; Ghouri et al., 2023). The relevance of autotetraploid rice for breeding is suggested by these studies. However, low fertility and low seed set have been frequently noted in autotetraploid rice throughout its known history compared to diploid rice. This problem has been a barrier preventing its utilization, leading to the slow progress of autotetraploid rice breeding research (Cai et al., 2007; Shahid et al., 2010; Chen et al., 2018; Li et al., 2018; Ghouri et al., 2019; Yang et al., 2019; Wu et al., 2019; Koide et al., 2020; Chen et al., 2021). After years of work, neo-tetraploid rice and tetraploid PMeS lines have recently been generated with high and stable seed setting characteristics (Chen et al., 2022). These rice materials are useful resources for the study of fertility-related molecular mechanisms and also the breeding application of tetraploid rice (Cai et al., 2007; He et al., 2011; Guo et al., 2017; Bei et al., 2019; Chen et al., 2019; Xiong et al., 2019; Ghaleb et al., 2020; Kamara et al., 2021; Liu et al., 2022).

The innermost anther cell layer, or tapetum, offers a secure environment, necessary nutrients, and components for developing microspores (Ariizumi and Toriyama, 2011). From male-sterile rice lines, several genes crucial for the growth of tapetum cells have been cloned. *EAT1/DTD* (Ji et al., 2013), *TIP2/bHLH142* (Fu et al., 2014; Ko et al., 2014), *UDT1* (Jung et al., 2005), *TDR* (Li et al., 2006), which encode *bHLH* transcription factor. R2R3-MYB family protein is encoded by *OsTDF1* (Cai et al., 2015). A PHD-finger protein that plays a role in the late tapetum development stage is encoded by the gene *PTC1* (Li et al., 2011), *NY1*, *SUBSrP1* and *DPS1*, were needed for panicle and anther development of rice (Zafar et al., 2020; Ali et al., 2022; Kamara et al., 2022);. Early rice tapetum development is significantly influenced by these genes, and mutations in these genes showed abnormal tapetal cells and male sterility. The development of pollen in autotetraploid rice is thought to be significantly influenced by the transcription factor LOC_*Os04g47890*, a member of the myeloblastosis (MYB) family (Li et al., 2018). Recently, *LOC_Os04g47890*, a MORE FLORET 1 MYB transcription factor, was reported to control spikelet development and was named *MOF1* (Ren et al., 2020). Understanding the regulatory mechanisms involving the tapetum and the expression levels of genes associated with the pollen wall is crucial for understanding pollen formation because the aforementioned genes are expressed there. All these genes were edited and investigated in diploid rice, but little is known in tetraploid rice.

RNA-Seq has the potential to identify differentially expressed genes with a greater expression level dynamic range (Wang et al., 2009). Many transcriptome investigations of plants, e.g., rice, maize, wheat, and Arabidopsis, etc., under various biotic and abiotic stresses, have been studied with efficacy (Ma et al., 2016; Vogel et al., 2016; Wang et al., 2016; Yadav et al., 2016). A reliable network of genes associated with pollen development has been utilized to learn more about how genes are regulated during the development of pollen activities of Arabidopsis, diploid, and polyploid rice, and insights from transcriptome analysis can be useful for identifying gene regulation during the development of rice pollen. Numerous studies have examined how autotetraploid rice develops its pollen (Wu et al., 2014; Wu et al., 2015; Li et al., 2016; Li et al., 2017). These RNA sequence data show the possibility of the genes controlling pollen development in tetraploid rice. However, the pollen fertility gene regulation network in tetraploid rice mutants remained largely unknown.

Our past investiagtion demostrated that the knock-out of *NY2* (*LOC_Os07g32040*), which encodes a eukaryotic translation initiation factor 3 subunit E, resulted in defective pollen, increased sterility, and straggled chromosomes in meiosis (Kamara et al., 2021). Here, we used anther transverse section analysis to observe cytological defects in anther pollen development between *ny2* and H1(wild type). The mutant *ny2* underwent abnormal tapetal cells, abnormal microspores, and a defective pollen wall formation. Moreover, a global gene expression study revealed that *ny2* alters the expression levels of pollen fertility-related genes, and qRT-PCR was employed to validate the expression patterns of these genes. Furthermore, between *ny2* and H1, reciprocal crosses were created, and normal fertility and an obvious advantage for seed setting were detected in F_1_. This study provided a new understanding of the genetic basis for pollen fertility and development in neo-tetraploid rice.

## Materials and methods

### Plant materials

Huaduo1 (H1), a neo-tetraploid line having high fertility that our lab developed, was used as a wild type. The *ny2* mutant was developed by CRISPR/Cas9 knock-out technology. F_1_ hybrids were created by reciprocal crosses between H1 and *ny2*. Rice plants were grown at the farm of South China Agricultural University, Guangdong.

### Anther development and pollen fertility observations of *ny2* mutant and wild type plants

The pollen fertility was determined based on our earlier research (Kamara et al., 2021). Under a microscope, the mature pollens were stained with 1% I_2_-KI (Motic BA 200, Motic, Xiamen, China).

The anther transverse section analysis was carried out according to (Kamara et al., 2022). The anthers of *ny2* mutants and wild-type (H1) plants were analyzed at various stages of anther development. First, the anthers were fixed in a solution of FAA (89% ethanol, 6% acetic acid, and 5% methyl aldehyde) for 48 hours at room temperature. After that, they were rinsed with 70% ethanol three times and dissected for semi-thin sectioning. A series of ethanol solutions were then used to dehydrate the anthers (80%, 90%, and 95%) for 30 minutes each. Finally, the tissues were processed according to the manufacturer’s protocol (Heraeus Kulzer) for Technovit 7100 Histological Analysis (Microtomy for Soft Tissues) at room temperature. Cross-sections of the anthers with a thickness of 3 μm were cut using a rotary microtome (Leica RM2235), stained with 1% toluidine blue, and sealed with neutral balsam. After that, the samples were examined and captured on camera with a microscope (Motic BA 200, China). The meiosis stage plays an important role in deciding the male fertility of rice. In this study, the frequency of tapetum abnormalities was observed during meiosis in *ny2* mutant with an anther floret length range from 4.9 - 5.2 mm (**Supplementary Table 1**).

### Analysis of the seed set of the F_1_ hybrids

In order to analyze the seed set of F_1_ hybrids generated by crossing H1 with *ny2*, 10 plants of each population were investigated at maturity. The selfing of F_1_ populations of all hybrids developed the F_2_ generations. The criteria for evaluating these characteristics were based on Guo et al. (2017). Data analysis was performed according to Kamara et al. (2022).

### RNA-seq analysis

The CRISPR/Cas9 editing system created a knock-out mutant of *ny2* in the neo-tetraploid line Huaduo1 (H1). The mutant lines were grown in the field, and the T_1_ and T_2_ mutant plants were sequenced. We further selected the homozygous mutant of *ny2* to observe the pollen development process. The anthers of H1 and T_3_ transgenic *ny2* lines (homozygous mutant) were gathered at the meiotic stage and kept at -80°C in three biological replicates for RNA isolation. Total RNA of each sample was extracted according to Chen et al. (2018). The variations in gene expression between the samples were recorded according to Kamara et al. (2022).

### Real-time qRT-PCR analysis

A group of 13 DEGs (Differentially Expressed Genes) was chosen randomly for the validation of RNA-Seq data through qRT-PCR. Primer Premier 5.0 software was utilized to design gene-specific primers (**Supplementary Table 2**). The Roche Transcriptor First Strand cDNA Synthesis Kit was used to perform the reverse transcription. The qRT-PCR experiment was executed according to the procedure defined by Kamara et al. (2022). The gene expression patterns were estimated by 2^-ΔΔCt^ method (Livak and Schmittgen, 2001). Every qRT-PCR test was run in triplicate.

## Results

### Cluster analysis and DNA variations of *NY2* (*LOC_Os07g32040*)

**Figure 1:**
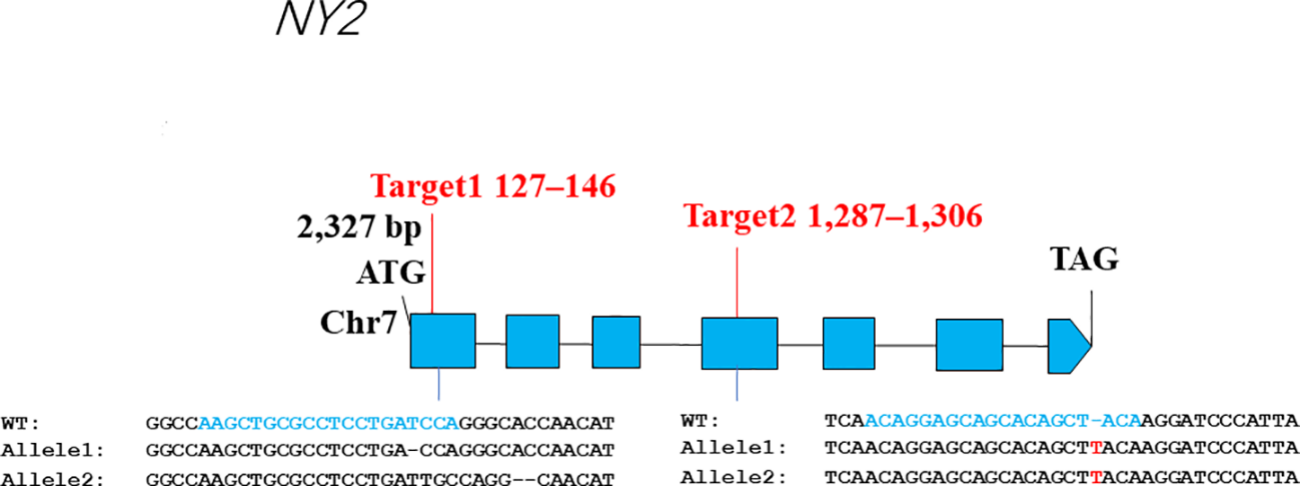
The gene structure and the CRISPR/Cas9 target sites of *LOC_Os07g32040* (*NY2*) gene. The other side of the figure shows the alignment of *ny2* sequences and wild-type (WT) having the CRISPR/Cas9 target sites.

The genomic sequence length of *NY2* is 2327 base pairs (bp), and its CDS sequence is 1311 bp, and it encodes a eukaryotic translation initiation factor 3 subunit E, with 426 amino acids (aa) (**Figure 1**). A resequencing data set of 121 rice accession was used to analyze the diploid, autotetraploid, and neo-tetraploid rice’s DNA variants in the NY2 sequence. Seventeen mutations were identified in the aforementioned materials, and 13 mutant sites in CDS (**Supplementary Table 3**; **Supplementary Figure 1**). Numerous frameshift mutations were found in *NY2*, including mutants 5 and 6 (**Supplementary Figure 2**). The majority of neo-tetraploid lines clustered with their parent, T45, according to cluster analysis, suggesting that T45 might be associated with the high fertility rice gene (**Supplementary Figure 3**).

### Genetic and phenotypic analysis of *ny2* mutant

**Figure 2:**
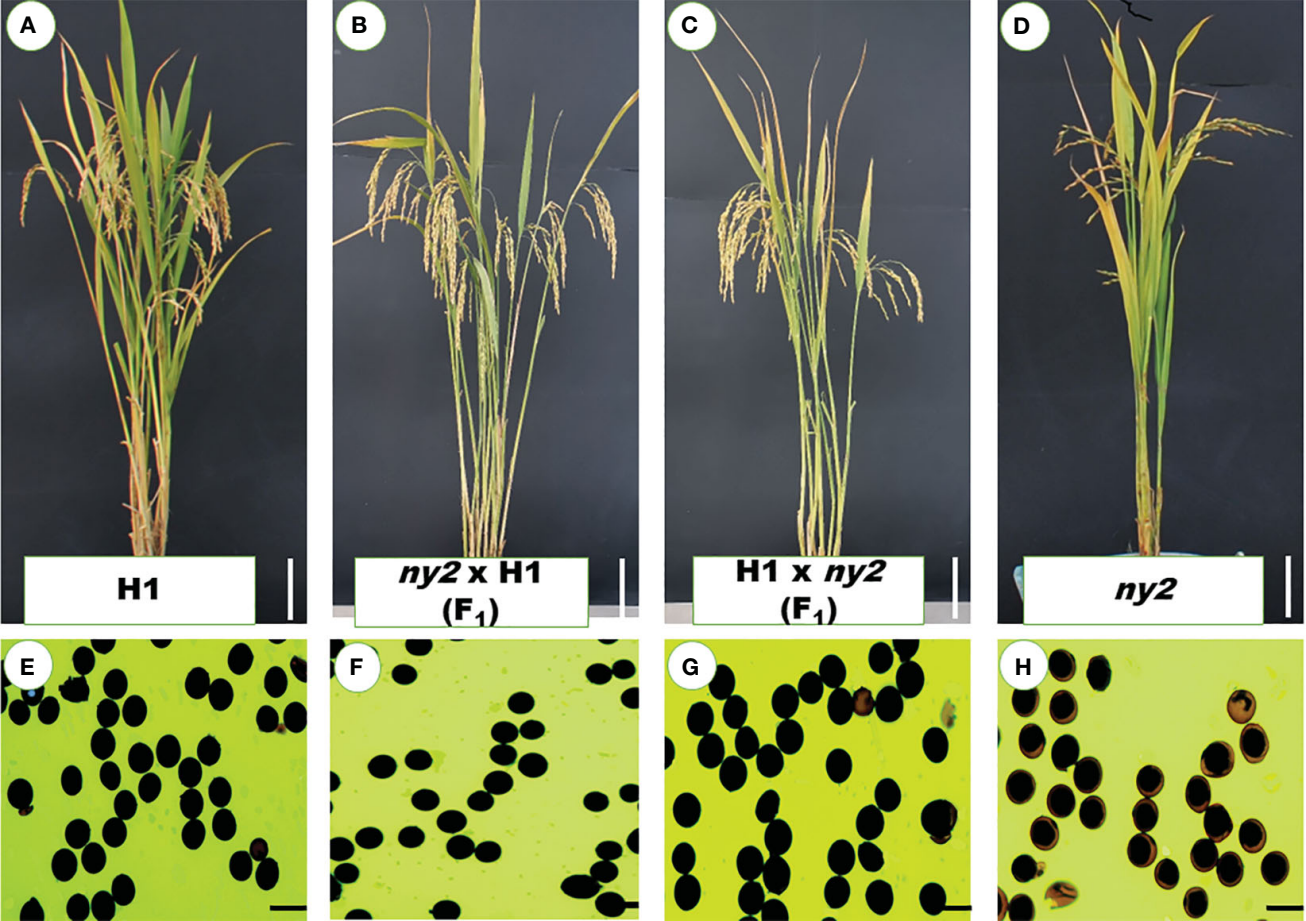
Hybrid lines’ and their parents’ phenotypic characteristics. **(A–D)** Plant morphology of H1, *ny2* x H1, H1 x *ny2* and *ny2* at the mature stage. Bars = 10 cm. **(E–H)** Pollen fertility of H1, *ny2* x H1, H1 x *ny2* and *ny2*. Bars = 50 μm.

To determine whether the *ny2* mutation caused the transgenic lines’ male-sterile phenotype, reciprocal crosses were made between *ny2* and H1. The F_1_ generation displayed normal plant types, pollen fertility, and obvious advantage for seed setting rate, just as the H1 (**Figures 2A–H**; **Table 1**), which is in contrast with the *ny2* mutant (**Figure 2D**; **Table 1**). In F_2_ plants, the phenotypes were studied, and the *ny2* mutant showed low seed set and pollen fertility (results not shown).

**Table 1:** Seed set and pollen fertility of F_1_ hybrids, H1 (WT), and *ny2*.

Genotypes	No. of plants	Pollen fertility (%)	Seed set rate (%)
H1 (WT)	10	92.31 ± 2.01a	79.75 ± 3.46a
*ny2* x H1 (F_1_)	10	92.69 ± 3.44a	78.71 ± 4.42a
H1 x *ny2* (F_1_)	10	91.09 ± 1.48a	76.78 ± 0.77a
*ny2*	10	46.01 ± 7.52b	45.34 ± 6.97b

The multiple comparison tests for each trait employed the least significant difference (LSD). Different letters indicate distinctions between the two samples that are statistically significant (p-value < 0.01).

### Cytological analysis of anther development in *ny2* mutant

**Figure 3:**
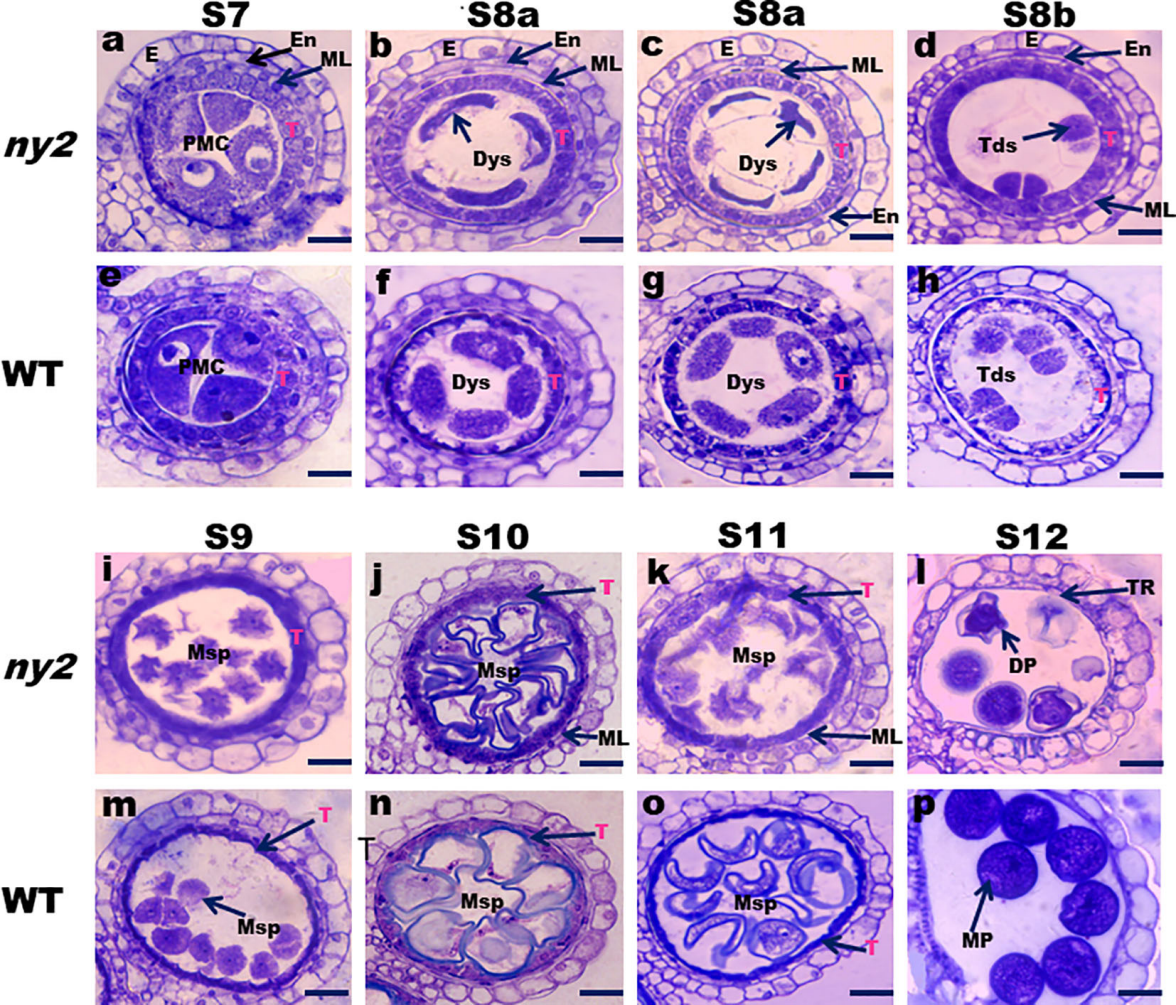
Transverse sections of anthers showing defective tapetal cell and microspore development in *ny2* mutant. Stage 7 **(A, E)**, Stage 8a **(B, C, F, G)**, Stage 8b **(D, H)**, Stage 9 **(I, M)**, Stage 10 **(J, N)**, Stage 11 **(K, O)**, and Stage 12 **(L, P)**. En, endothecium; E, epidermis; T, tapetum; PMC, pollen mother cells; ML, middle layer; Dys, dyad cells; DP, deformed pollen; Tds, tetrads; MP, mature pollen; TR, tapetal remnants Msp, microspore; Bars=20 μm **(A–P)**.

A cross-section of the *ny2* and WT anthers at diverse pollen development phases was analyzed according to Kamara et al. (2022) to unveil the cytological abnormalities in anther pollen development. Stage 7 transverse sections of *ny2* and WT anthers showed no discernible differences, and both showed a typical structure of all layers (**Figures 3A, E**). At stage S8a, the pollen mother cells were abnormal in the *ny2* mutant compared to WT. The middle layer cells became narrower and more degenerated, and the tapetum cells of the WT exhibited high degeneration and became less vacuolated when compared with *ny2* mutant anthers at stage S8a (**Figures 3B, C, F, G**). At stage S8b, middle layer cells and tapetum degenerated, and the pollen mother cells form tetrads. In contrast, the *ny2* mutant anthers formed abnormal tetrads, the middle layer cells became more visible, and the tapetum became highly vacuolated (**Figures 3D, H**). During stage 9, WT tapetum cells turned thin, and the microspores were normal, but the tapetum of *ny2* remained vacuolated, and the microspore cells were abnormal and significantly degenerated (**Figures 3I, M**). At stage 10, the WT tapetum cells shrunken further, the middle layer disappeared, and the microspores were fully developed in the WT circular anther locules. Still, the tapetum cell of *ny2* was vacuolated, the middle layer remained obvious, and microspore cells were abnormal and degenerated (**Figures 3J, N**). During stage 11, the WT tapetum cell became less vacuolated, and the middle layer disappeared. In contrast, the microspore cells of *ny2* were seriously affected and showed severe degeneration, the tapetum cell was normal, and the middle layer stayed visible at stage 11 (**Figures 3K, O**). At stage 12, the WT anther produced round, starchy pollen grains that had been fully stained. The pollen grains in *ny2* were distorted and encircled by leftover tapetum fragments, which eventually caused male sterility and pollen abortion (**Figures 3L, P**).

### Comparative transcriptome analysis in wild type (H1) compared with *ny2* during PMC meiosis

The meiosis stage plays vital role in deciding the male fertility of rice. Hence, transcriptome analysis was used to investigate the patterns of gene expression during the meiosis stage in WT compared with *ny2*. Approximately 49,077,837 clean reads of high quality were found in each sample, which is 92.09% of the total 53,266,124 reads. When the clean reads were mapped onto the Nipponbare reference genome, 91.49 to 92.69% of the reference genome’s annotated transcripts were obtained in WT instead of *ny2* (**Table 2**). The percentage of Q30 base was 93.47% or more, and the GC content ranged from 48.44% to 50.49% in all samples (**Table 2**). The correlation among the three replications of each sample varied from 0.77 to 0.98, suggesting the consistency of the three replicates (**Supplementary Table 4**).

**Table 2:** Overview of quality reads in WT compared with *ny2* during meiosis.

Sample	Total Reads	Clean reads	Mapped Reads Rate	Unique-Mapped Reads Rate	GC Content	Q30
WT-1	53,928,296	49,935,957	92.60%	89.45%	50.15%	93.67%
WT-2	58,998,470	54,685,039	92.69%	89.61%	50.49%	93.79%
WT-3	66,336,078	61,239,699	92.32%	88.93%	50.44%	93.86%
*ny2-1*	44,260,124	40,585,269	91.70%	83.14%	48.44%	93.83%
*ny2-2*	47,429,400	43,391,913	91.49%	82.92%	49.49%	93.47%
*ny2-3*	48,644,376	44,629,144	91.75%	83.32%	48.58%	93.55%

**Figure 4:**
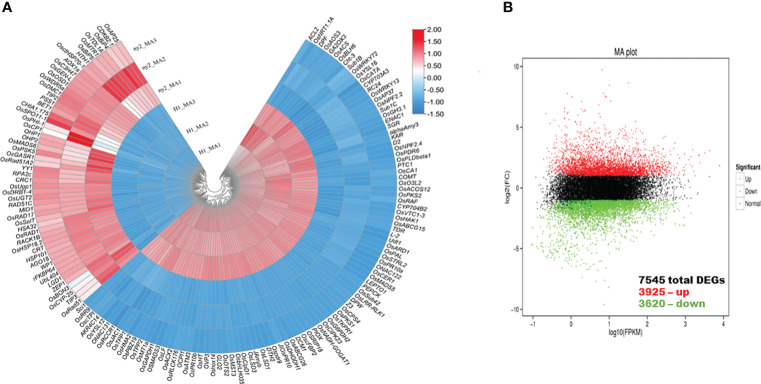
Sliding window and MA plot of the differential gene expression patterns in wild type (H1) compared with *ny2.* **(A)** The sliding window plot presenting the differentially expressed genes (DEGs) in wild type (H1) compared with *ny2*. **(B)** DEGs in wild type (H1) compared with *ny2.* The threshold used to assess the significance of gene expression differences was P<0.05.

A total of 7545 genes exhibited differential expression levels during meiosis in WT compared with *ny2* (**Figure 4B**; **Supplementary Table 5**). Among these DEGs, 3925 and 3620 genes exhibited up- and down-regulation (**Figure 4B**; **Supplementary Table 5**). These results revealed that numerous genes were expressed during PMC meiosis in *ny2*. However, an abundance of key DEGs were highly expressed in H1 than *ny2* mutant (**Figure 4A**). We also subjected the significant genes to the Kyoto encyclopedia of genes and genomes (KEGG) pathway and gene ontology (GO) enrichment analysis.

Gene ontology (GO) enrichment analysis of the 7545 DEGs depicted 210 and 120 GO terms that were significantly down- and up-regulated in wild type (H1) compared with *ny2*, respectively. Cellular and metabolic processes had the highest percentage of representation in the biological process category. Binding and catalytic processes dominated the category of molecular function, while cells and cell parts dominated the category of cellular component (**Supplementary Figure 4**). We further characterized the GO terms related to down-regulated DEGs in *ny2* in the biological process (**Supplementary Table 6A**). Of all these enrichment terms, some associated with anther development were known as significant ones. Of the down-regulated DEGs, disaccharide metabolic process, cellular carbohydrate metabolic process, and glycoside metabolic process were detected among the important enrichment terms, and the gene *OsSSIIIa* (*LOC_Os08g09230*) belonging to cellular carbohydrate metabolic process term participates in the glucan biosynthetic process and starch synthase activity. The gene *RSUS2* (*LOC_Os06g09450*), belonging to the glycoside metabolic process term participated in the biosynthetic process, sucrose synthase and sucrose metabolic process, and the gene *RSUS3* (*LOC_Os07g42490*), belonging to the disaccharide metabolic process term participated in the biosynthetic process, sucrose synthase and sucrose metabolic process (**Supplementary Table 6A**). It’s interesting to note that the GO term for the metabolism of carbohydrates was significantly enriched in both down- and up-regulated genes, and four DEGs *Osg1* (*LOC_Os01g71930*), *OsHXK7* (*LOC_Os05g09500*), *OsHXK9* (*LOC_Os01g52450*) were down-regulated, and *OsSUS7* (*LOC_Os04g17650*) was up regulated were identified to play a significant role in anther pollen development (**Table 3**; **Supplementary Tables 6A, B**). Except for the four important terms above, some other terms, including pollination, recognition of pollen, pollen-pistil interaction, reproduction, reproductive process, oligosaccharide metabolic process, carbohydrate biosynthetic process, oligosaccharide biosynthetic process, cellular lipid catabolic process, were represented in the down-regulated gene class transcripts (**Supplementary Table 6A**).

**Table 3:** Genes involved in the metabolism of sucrose and starch showed downregulation in *ny2*.

Name	Gene ID	Description	*ny2*
*RSUS2*	*LOC_Os06g09450*	sucrose synthase gene	Down
*RSUS3*	*LOC_Os07g42490*	sucrose synthase gene	Down
*OsHXK7*	*LOC_Os05g09500*	hexokinase gene	Down
*OsHXK9*	*LOC_Os01g52450*	hexokinase gene	Down
*OsSWEET4*	*LOC_Os02g19820*	sugar transport-related gene	Down
*Osg1*	*LOC_Os01g71930*	sugar degradation-related gene	Down
*OsMST3*	*LOC_Os07g01560*	monosaccharide transporter gene	Down
*OsSSIIIa*	*LOC_Os08g09230*	starch synthase gene	Down

**Figure 5:**
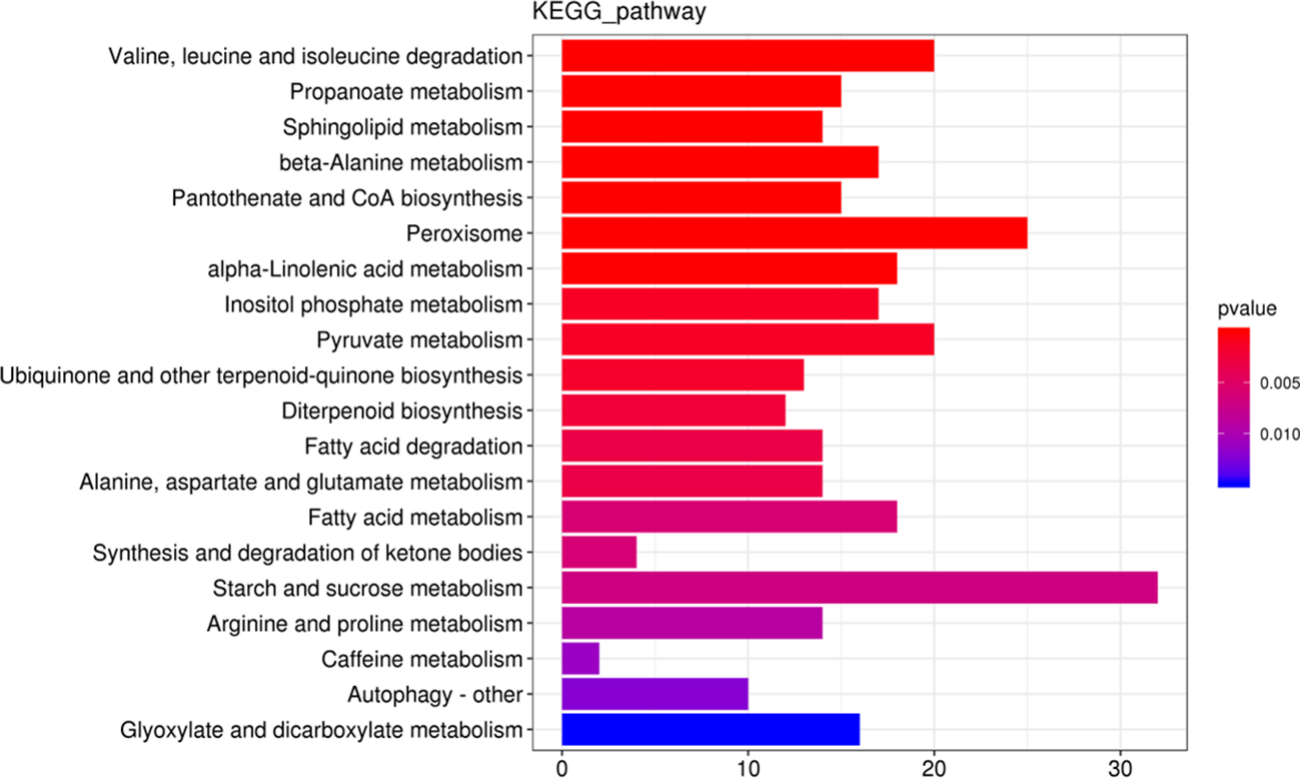
KEGG pathway analysis of the down-regulated DEGs in wild type (H1) compared with *ny2*. The abscissa represents the number of genes, the ordinate represents the enrichment pathways, the column length represents the number of genes, and color represents the *p*-value.

We mapped all DEGs to their various KEGG enrichment analyses to get a deeper perception of the specific pathways that control the biological processes mentioned above. The most significant biological pathways include protein processing in the endoplasmic reticulum, ribosome, and oxidative phosphorylation (**Supplementary Figure 5**). We further analyzed the down-regulated DEGs in WT compared with *ny2*. Among the top 20 most enriched down-regulated pathways, starch and sucrose metabolism (ko00500) and peroxisome (ko04146) were the most significantly down-regulated pathways in *ny2* (**Figure 5**). Except for the two important pathways above, some other relevant biological pathways related to anther development, such as pyruvate metabolism (ko00620), fatty acid metabolism (ko01212), sphingolipid metabolism (ko00600) and fatty acid degradation (ko00071), were also recognized in the down-regulated DEGs. For example, starch and sucrose metabolism pathway DEGs were related to the synthase of sucrose, sucrose transporter, sucrose invertase, hexokinase, and starch. Sucrose and invertase are broken down into monosaccharides that are conveyed to the cells and utilized for starch biosynthesis (Ruan et al., 2010; Ruan, 2012). Two hexokinase genes (*OsHXK7* and *OsHXK9)* and two sucrose synthase genes (*RSUS2* and *RSUS3*) showed down-regulation in *ny2*. Saccharides are transported from source to sink by sucrose and monosaccharide transporters (Smeekens, 2000; Wang et al., 2008). *OsSWEET4*, a sucrose transporter gene and *OsMST3*, a monosaccharide transporter gene, exhibited down-regulation in *ny2*. In addition, a sugar degradation-related gene (*Osg1*) and a starch synthase gene (*OsSSIIIa*) were found to be down-regulated in *ny2* (**Table 3**). *LOC_Os03g07140*, a *DPW* gene that belongs to fatty alcohol synthesis and peroxisome pathway, plays a vital role in pollen sporopollenin biosynthesis and anther cuticle in rice also showed down-regulation in *ny2*.

### Down-regulation of DEGs relates to pollen fertility in wild type (H1) compared with *ny2*

In previous studies, reduction of pollen fertility gene expression was frequently observed, which was thought to be the main cause of autotetraploid rice’s low pollen fertility (Wu et al., 2014; Li et al., 2018; Kamara et al., 2022). This study examined the genes at pollen mother cell (PMC) meiosis stage using comparative transcriptome profiling. We compared the pollen fertility-related genes with the DEGs detected in the present study (Fujita et al., 2010; Deveshwar et al., 2011; Yant et al., 2013; Wright et al., 2014; Wu et al., 2014) and found nine important down-regulated genes associated with tapetum or pollen fertility during meiosis in wild type (H1) compared with *ny2*, including *LOC_Os10g35180*, *LOC_Os04g51070*, *LOC_Os09g27620*, *LOC_Os06g36080*, *LOC_Os11g47809*, *LOC_Os08g03682*, *LOC_Os02g02820*, *LOC_Os03g15710*, and *LOC_Os07g36460*. Of these genes, *OsABCG26* (*LOC_Os10g35180*) is an ABC transporter; its mutant displayed defects in tapetal cells and caused pollen sterility in rice (Chang et al., 2016). *EAT1* (*LOC_Os04g51070*) is a programmed cell death regulatory gene, bHLH transcription factor, and delayed tapetum degradation during rice pollen development (Niu et al., 2013). A PHD-finger protein called *TMS9-1 (LOC_Os09g27620)*, also referred to as *PTC1* and *OsMS1*, is a thermosensitive male sterility gene that regulates pollen and tapetal cell development in rice (Qi et al., 2014; Yang et al., 2019; Wu et al., 2020). *KIN14M* (*LOC_Os06g36080*) is a kinesin motor domain-containing gene, and reduced pollen fertility and decreased seed setting in neo-tetraploid rice are the effects of this gene’s loss of function (Yu et al., 2020). *OsMT1a* (*LOC_Os11g47809*) is a metallothionein gene, and the knock-out of this gene causes pollen sterility (Lu et al., 2020). *CYP703A3* (*LOC_Os08g03682*) cytochrome P450 hydroxylase gene that regulates tapetum and male fertility in rice (Yang et al., 2014). A tapetum development regulatory gene known as *TDR (LOC_Os02g02820)*, its mutant plants showed tapetum and middle layer degeneration retardation and microspore collapse (Li et al., 2006). *OsSTRL2* (*LOC_Os03g15710*), an atypical strictosidine synthase, which is highly expressed in microspores and tapetal cells, and its mutants cause male abortion (Zou et al., 2017) and *UDT1* (*LOC_Os07g36460*) is a *bHLH* transcription factor, and a tapetum development regulatory, knock-out mutant showed defects in tapetal cells and caused male sterility in rice (Jung et al., 2005) (**Table 4**).

**Table 4:** Down-regulated genes associated with pollen fertility in *ny2*.

Gene name	Function	Mutant phenotype	Reference
*OsABCG26*	ATP Binding Cassette G26	defects tapetal cells and pollen sterility	Chang et al., 2016
*EAT1*	bHLH transcription factor	tapetum cell death and pollen sterility	Niu et al., 2013
*TMS9-1*	thermosensitive male sterility	tapetal cell death and pollen sterility	Wu et al., 2020; Qi et al., 2014
*KIN14M*	kinesin motor	decreased pollen fertility and seed set	Yu et al., 2020
*OsMT1a*	Metallothionein	defects tapetal cells and male sterility	Lu et al., 2020
*CYP703A3*	cytochrome P450 hydroxylase	developmental defects of cuticle on anther surface and male fertility	Yang et al., 2014
*TDR*	tapetum development regulatory	tapetum degradation and pollen sterility	Li et al., 2006
*OsSTRL2*	Atypical strictosidine synthase	male sterility	Zou et al., 2017
*UDT1*	tapetum development regulatory	defects tapetal cells and male sterility	Jung et al., 2005

### Candidate genes expression analysis in wild type (H1) compared with *ny2*

**Figure 6:**
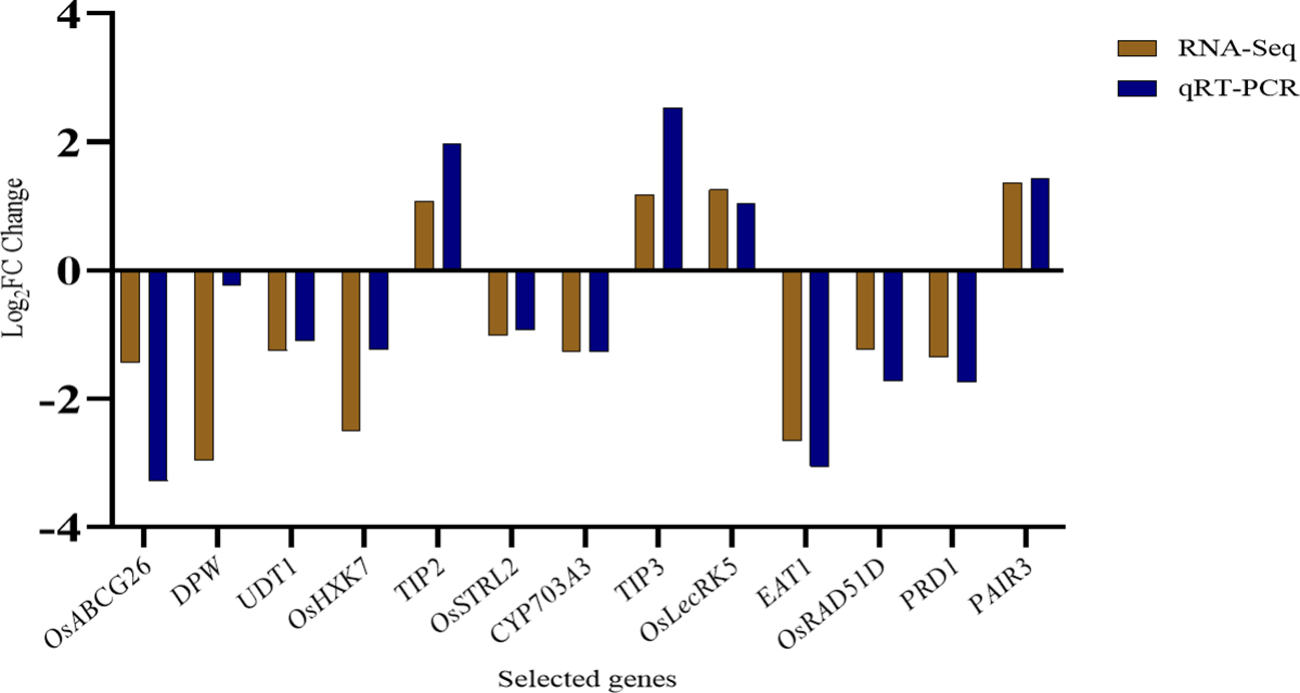
Comparison of the log_2_ (FC) of thirteen selected genes using qRT-PCR analysis in wild type (H1) compared with *ny2*.

To confirm the gene expression levels of important genes in wild type (H1) and *ny2*, a subset of 13 tapetum or pollen fertility and transcription factor-associated genes were selected for qRT-PCR analysis. We contrasted the qRT-PCR results with the ones from the RNA-Seq analysis of these transcripts. The RNA-Seq analysis and expression trends were in agreement, with a correlation coefficient of R^2 = ^0.7949 (**Figure 6**; **Supplementary Figure 6**).

### Transcriptional factors (TFs) associated genes showed down-regulation in wild type (H1) compared with *ny2*

**Figure 7:**
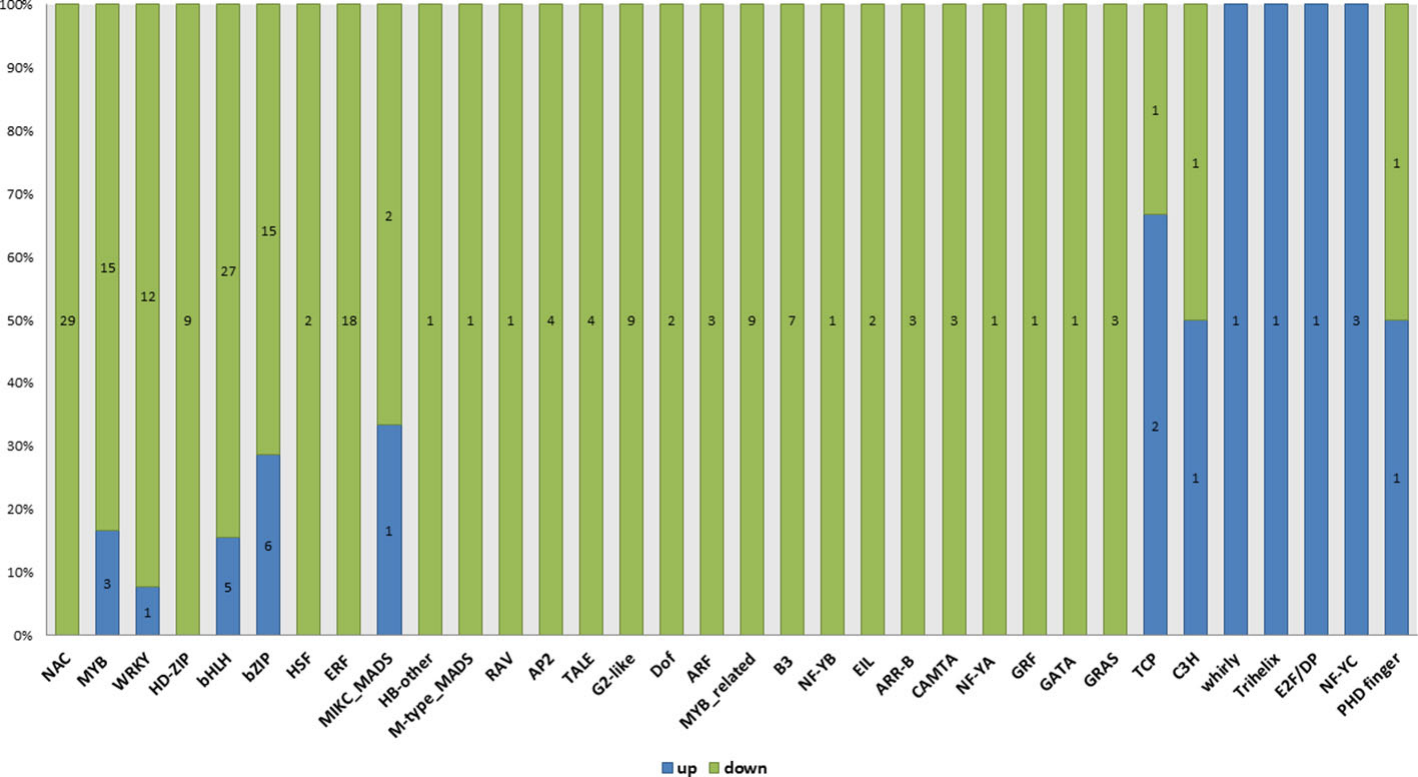
Differentially expressed genes associated with different transcription factor families discovered in WT compared with *ny2*. We discovered 212 putative TF DEGs, which were categorized into 34 groups.

Transcription factors are evident in controlling varied physiological activities in response to fertility. Transcription (GO: 0006350), regulation of transcription (GO: 0045449) and transcription regulator activity (GO: 0030528) were the significant GO terms enriched only in WT compared with *ny2*. In all, 212 DEGs were identified as transcription factors belonging to 34 (TFs) groups in wild type (H1) compared with *ny2*. Of the transcription factor related genes, 189 displayed down-regulation (**Figure 7**; **Supplementary Table 7**). The largest groups reported were transcription factor DEGs of NAC, bHLH, bZIP, MYB and ERF. Of these groups, bHLH, MYB family, MIKC_MADS and PHD-finger transcription factor were shown to participate in the pollen development process in previous studies (Zhang et al., 2010; Li et al., 2011; Sui et al., 2015; Zhang et al., 2015; Zhu et al., 2015; Ranjan et al., 2017; Xiang et al., 2020). Here, we identified 13 transcription factors related to genes that regulate pollen fertility in rice. Among these DEGs, *EAT1, UDT1, TDR, TP2, OsbHLH138*, and *OsPIL16* encode the bHLH family transcription factor, *PTC1* and *TIP3* encode a PHD-finger transcription factor *OSMADS3, OsMADS5* and *OsMADS13* encode MIKC_MADS family protein, *OsRR24* is an ARR-B transcription factor, and *LOC_Os08g06370* is a MYB family transcription factor. Among these genes, *EAT1*, *UDT1*, *TDR*, *OsbHLH138*, *OsPIL16*, *OsMADS3*, *OsMADS5*, *OsRR24* and *PTC1* were down-regulated and *LOC_Os08g06370*, *TIP, OsMADS13*, and *TIP3* were found to be up-regulated (**Figure 7**).

## Discussion

### *NY2* may regulate tapetal cell, microspore and middle layer development in tetraploid rice

The precise control of tapetal cell death is vital for anthers and pollen development in rice. Timely initiation of tapetal programmed cell death (PCD) is crucial for releasing pollen wall materials, such as carbohydrates, lipid molecules, and other related nutrients, from the tapetum cells and transporting them to the anther and microspore surface for deposition (Li et al., 2011). Many DEGs, including *EAT1*, *UDT1*, *TDR*, *OsABCG26*, *PTC1*, *OsSTRL2*, *SUBSrP1*, *DPS1*, and *NY1* (Jung et al., 2005; Li et al., 2011; Niu et al., 2013; Chang et al., 2016; Zou et al., 2017; Zafar et al., 2020; Ali et al., 2022; Kamara et al., 2022), play critical controls over the development of the tapetum PCD and microspores. Silenced or loss of function of these genes delays tapetal cell degradation and promotes abnormal microspore development and pollen abortion. In autotetraploid rice, tapetal cells and microspore abnormalities were observed to be the principal causes of pollen abnormality (Li et al., 2018; Lu et al., 2020; Wu et al., 2020; Chen et al., 2022; Kamara et al., 2022). Similarly, in this study, the cytological analysis showed that the *ny2* mutant displayed defects in tapetal cells, microspores and pollen wall development, which caused pollen sterility and male abortion.

The RNA-seq analysis revealed that 7545 genes displayed differential expression patterns during meiosis. Of these DEGs, 3925 and 3620 were up- and down-DEGs. Among the down-regulated genes, the expression levels of several key DEGs involved in tapetum or pollen fertility were significantly altered in the *ny2* anthers. For example, *OsABCG26* (*LOC_Os10g35180*) is an ABC transporter; its mutant plant displayed defects in tapetal cells and caused male sterility in rice (Chang et al., 2016). *EAT1* (*LOC_Os04g51070*) is a programmed cell death regulatory gene, bHLH transcription factor; its mutant delayed tapetum degradation in rice (Niu et al., 2013). The rice gene *TMS9-1 (LOC_Os09g27620*), also referred to as *PTC1* and *OsMS1*, is a PHD-finger protein that regulates tapetal cell and pollen fertility (Li et al., 2011; Qi et al., 2014; Yang et al., 2019; Wu et al., 2020). *KIN14M* (*LOC_Os06g36080*) is a kinesin motor gene, and the knock-out of *KIN14M* showed a decrease in pollen fertility and seed setting in neo-tetraploid rice (Yu et al., 2020). *TDR* (*LOC_Os02g02820*) is a tapetum development regulatory gene; its mutant plants exhibited abnormal tapetum, middle layer and collapse of microspores (Li et al., 2006). *OsSTRL2* (*LOC_Os03g15710*) is an atypical strictosidine synthase highly expressed in microspores and tapetal cells. Its mutant showed abnormal fertility (Zou et al., 2017), and *UDT1* (*LOC_Os07g36460*) is a tapetum development regulatory gene; its mutant showed defects in tapetal cells and ultimately resulted in pollen sterility in rice (Jung et al., 2005). Similar to these findings, a number of past studies showed that the down-regulated genes are the main cause of pollen abortion in tetraploid rice (Wu et al., 2014; Wu et al., 2017; Chen et al., 2019; Kamara et al., 2022). Our findings suggested that like the nine published tapetum or pollen fertility genes mention-above, *NY2* is required for normal Pollen formation by regulating tapetal cells, microspore and pollen wall development in neo-tetraploid rice.

### Alters expression in starch and sucrose metabolism-associated genes cause pollen sterility in *ny2* mutant

Energy from carbohydrates is needed to maintain the growth of the anthers and pollen, but they also serve as a growth indicator (Clement et al., 1996). The regulation of carbohydrates is actively regulated by many related proteins or genes. Numerous studies have shown that altered gene or protein expression in anthers impairs pollen development and adversely affects pollen fertility. Plant anthers possess the greatest sink capacity in flower, to support early development, enormous amounts of sugars are arranged on the anthers (Clement et al., 1996; Castro and Clément, 2007). Sucrose transporters (SUTs) are vital for sucrose uptake in diverse stages of sugar translocation and uphold a link-up relationship between pollen development and sucrose transport (Srivastava et al., 2008; Hirose et al., 2010). In plants, sugars are manufactured in the mesophyll cells of developed leaves that are best known as source organs. Hence, precise sugar production, storage, and transportation are essential for sustaining growth and development in plants. Due to decreased sucrose transport in the phloem, mutations in the *AtSUC2* gene in the plant *Arabidopsis thaliana* cause growth and fertility reductions (Gottwald et al., 2000; Gould et al., 2012). *OsSWEET4*, *OsSWEET11*, or *OsSWEET14* mutants that were silenced or lost their ability to function exhibited low fertility, delayed reproductive traits, smaller seeds, and shriveled caryopses (Chu et al., 2006; Antony et al., 2010; Li et al., 2012; Ma et al., 2017). The suppression of *Osg1* causes male sterility (Wan et al., 2010). Therefore, all these results showed that sugar transporter or degradation and sucrose play an essential role in pollen formation. Here, the same sugar transporter gene (*OsSWEET4*) and (*Osg1*) were identified to be down-regulated in *ny2*. Sucrose synthase and sucrose invertase are important enzymes in sugar metabolism in plants (Ruan, 2012). Sucrose synthase genes (*RSUS3*) and (*RSUS2*) were significantly down-regulated, while the sucrose invertase gene (*OSINV4*) showed upregulation in *ny2*. Hexose, an essential energy source, plays an essential role in pollen development and forms an important component for cell wall synthesis. A deficiency in hexokinase *HXK5* disturbed the usage of starch in pollen grains, which resulted in pollen sterility (Lee et al., 2020). Here, hexokinase genes (*OsHXK7* and *OsHXK9)* showed down-regulation in *ny2*. In addition, Starch, a polymer of glucose residues, serves as the main energy source and carbon skeleton in developed pollen grains, enabling pollen germination and the development of pollen tubes for proper fertilization. Consequently, it is believed that deficiency of starch synthesis in pollen grains is the root of male sterility (Datta et al., 2002; Wu et al., 2016). As a result, modifying the starch contents of pollen grains has proven to be a new method of causing male abortion (Wu et al., 2016). In our study, a starch synthase gene (*OsSSIIIa*) was found to be down-regulated in *ny2.*

In pollen development, monosaccharide transporters play a crucial role (Wang et al., 2008). The development of rice pollen is evidently affected by the expressions of *OsMST5* and *OsMST8* (Ngampanya et al., 2003; Mamun et al., 2006). For example, *cas* mutant displayed remarkably lower expression levels of *OsMST8*, which reduced levels of carbohydrates and sugar in flower organs and led to pollen abortion (Zhang et al., 2010). Here, *OsMST3*, a monosaccharide transporter, was discovered to have decreased expression in *ny2*. Therefore, we speculated that the down-regulation of sucrose synthase, hexose, starch, and monosaccharide genes might be accountable for the defective pollen development in the *ny2* mutant.

### The down-regulation of transcription factors might cause male fertility in *ny2* mutant

In higher plants, male gametophytes play an important role in pollen fertility. The complex and precise control of a large number of transcription factors is required for the tight regulation of pollen grain development (Guan et al., 2014; Lei et al., 2017).

A complex regulatory network of various transcription factors (TFs) working in a tissue-specific manner is essential to the development of anthers. Given that one of the key applications of plant biotechnology is the genetic changes in the male reproductive system, understanding the TFs related to the regulatory network of anther development is crucial. During the growth and development of plants, changes in the expression levels of some transcription factors may result in pollen defects and male sterility. For instance, overexpressing *WRKY27* in *Arabidopsis* plants resulted in defects with pollen cracking, growth issues, and male sterility (Mukhtar et al., 2018). According to reports, *OsTDF1* and *OsMYB103* restored fertility in the *tdf1* and *ms188* mutants of *Arabidopsis*, respectively, and their gain- or loss-of-function induced male abortion in rice (Phan et al., 2012; Cai et al., 2015). *OsMYB103* (*OsMYB80*) controls the development of anther by regulating the middle layer development and tapetum in rice (Zhang et al., 2010). The down-regulation of *OsMYB106* causes pollen sterility in rice (Chen et al., 2014). Past studies also reported that knock-down and knock-out mutants of *bHLH142* resulted in male sterile plants in rice (Fu et al., 2014; Ko et al., 2014). Additionally, the unique rice BHLH transcription factor DTD works in tandem with TDR to control the development of the tapetum and pollen in rice (Ji et al., 2013). Here, we identified 9 down-regulated transcription factor DEGs associated with rice pollen fertility. Of these genes, *OsbHLH138* (*LOC_Os03g27390*) is a basic helix-loop-helix transcription factor, which controls thermos-sensitive male infertility in rice via activation of *TMS5* (Wen et al., 2019; Chen et al., 2022). *OsMADS3* (*LOC_Os01g10504*) is a MADS box gene, and the loss of function of its mutant causes male abortion in rice (Zhang et al., 2015). *OsRR24* (*LOC_Os02g08500*) is a Type B response regulator that encodes an ARR-B transcription factor; its mutant showed abnormal meiosis and tapetal cells in rice (Zhao et al., 2018). Mutant plants of these three genes showed abnormal tapetum development and pollen defects. In *the ny2* mutant, male sterility and a similar defect in the tapetum were also seen.

## Conclusions

In this study, the cytological analysis indicated that *ny2* underwent abnormal tapetal cells, microspores and middle layer development. Genetic analysis revealed that the F_1_ plants showed normal fertility and an obvious advantage for the seed set. Also, a large number of tapetum or pollen fertility, sucrose and starch metabolism and transcription factors related genes showed down-regulation. The regulatory gene network reported in this study may provide valuable information about the molecular mechanism related to fertility regulation in tetraploid rice

## Data availability Statement

The original contributions presented in the study are included in the article/**Supplementary Material**, further inquiries can be directed to the corresponding authors.

## Author contributions

MQS and JW. designed and conceived the experiments. NK, YJ, WH, L C, LZ, CZ, XH and FNS. performed the experiment and analyzed the data. NK, YJ, MQS and XL wrote the paper. XL. generated the neo-tetraploid and autotetraploid rice lines. All authors contributed to the article and approved the submitted version.
