# Supplementary material for: Comparative cytological and transcriptome analyses of ny2 mutant delayed degeneration of tapetal cells and promotes abnormal microspore development in neo-tetraploid rice
Source: Front Plant Sci. 2023 Jul 17;14:1229870. doi: 10.3389/fpls.2023.1229870 (PMC10387629; doi:10.3389/fpls.2023.1229870)
Supplement: Supplementary file 1 [file DataSheet_1.pdf]

Supplementary files:

Supplementary file: Figure S1. Analysis of variations in *NY2* sequence of 121 rice materials. **Figure S2**. The prediction of tertiary structure of mutant proteins. **Figure S3**. Cluster analysis of *NY2* in 121 rice materials. **Figure S4**. GO analysis of differentially expressed genes classification into three main categories, biological process, molecular function and cellular component. **Figure S5**. KEGG pathways enriched in wild type (H1) compared with *ny2* during meiosis. **Figure S6**. Comparison of the log2 (FC) of 13 selected genes using qRT-PCR analysis in WT compared with *ny2*. **Supplementary file 2: Table S1**. Floret length during meiosis in wild type (H1) and *ny2* (mutant) rice. **Table S2**. List of primers used for qRT-PCR analysis. **Table S3**. Mutation types of *NY2* in 121 sequencing rice materials. **Table S4**. Pearson correlation analysis of H1 (WT) compared with *ny2*. **Table S5.** Differentially expressed genes in wild type (H1) compared with *ny2* during meiosis. **Table S6a**. Significant up-regulated GO term of differentially expressed genes in wild type (H1) compared with *ny2* during meiosis. **Table S6b**. Significant down-regulated GO term of differentially expressed genes in wild type (H1) compared with *ny2* during meiosis. **Table S7**. Differentially expressed transcription factors in WT compared with ny2 during meiosis.

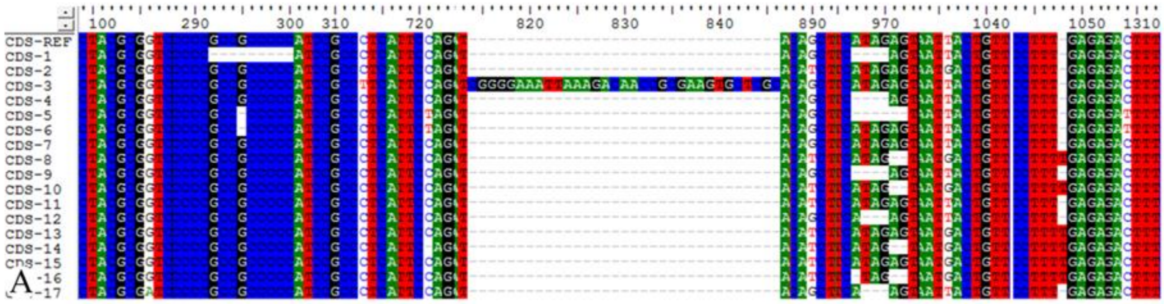

Supplementary file 1: Figure S1. Analysis of variations in *NY2* sequence of 121 rice materials

Note: white color indicates the 13 mutation sites, while CDS-1-17 represent mutation types compared to Nipponbare reference genome in different materials. Please see Supplementary Table S1 for mutation types.

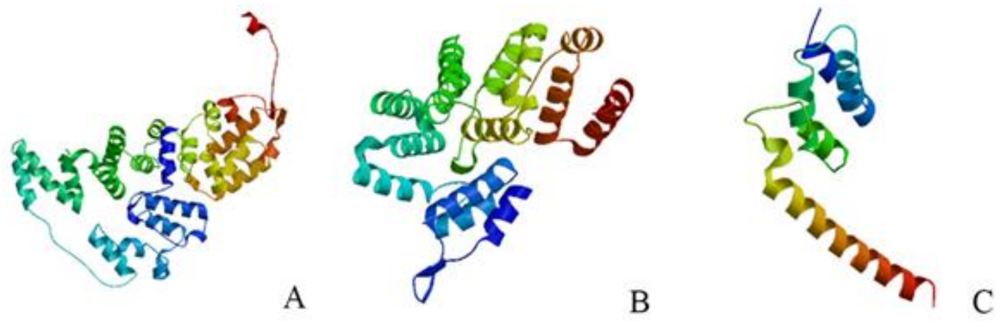

Supplementary file 1: Figure S2. The Prediction of tertiary structure of mutant proteins. (A) WT (reference genome, NIP). (B) Mutant 5. (C) Mutant 6

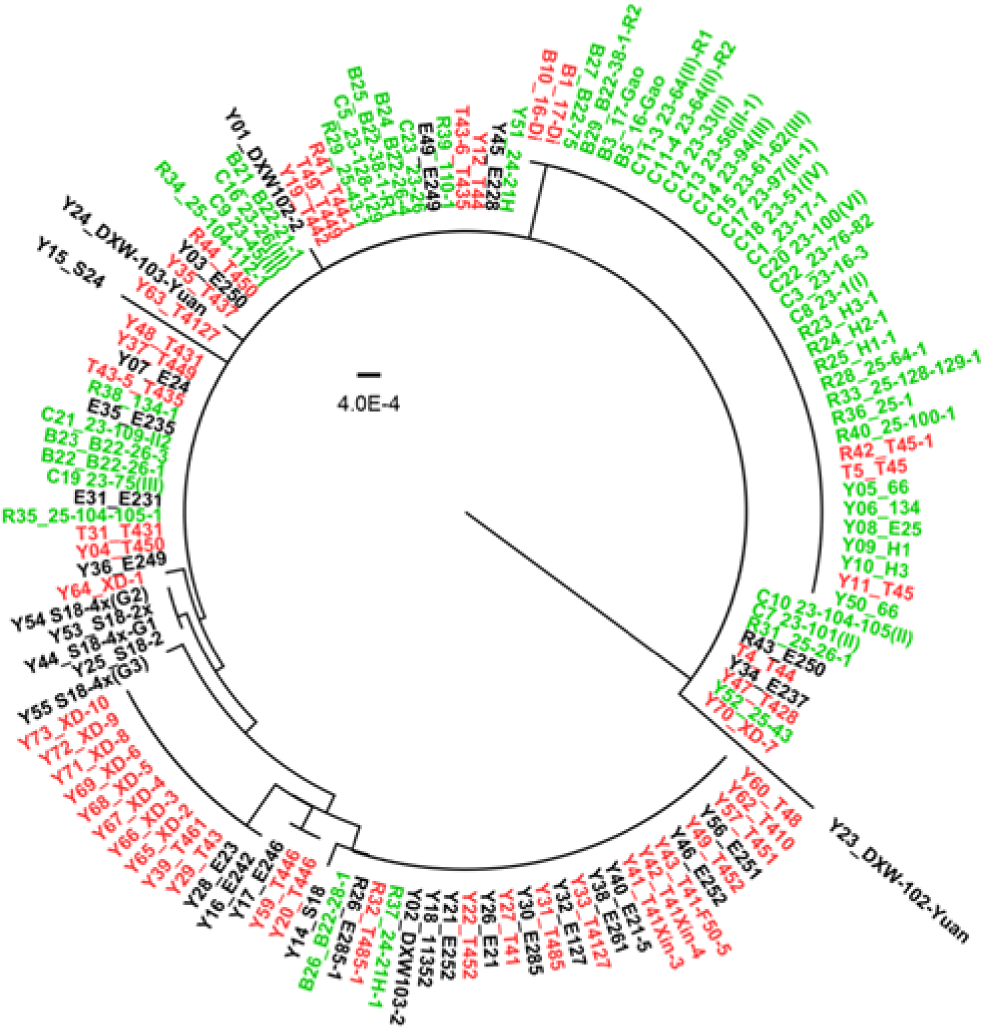

Supplementary file 1: Figure S3. Cluster analysis of *NY2* in 121 rice materials.

The rice lines in green are neo- tetraploid lines with high fertility; the rice lines in pink are autotetraploid lines with low fertility; and the rice lines in black are diploid lines with high fertility. Genome classification, population structure and types of some lines are mentioned in Yu et al., 2021.

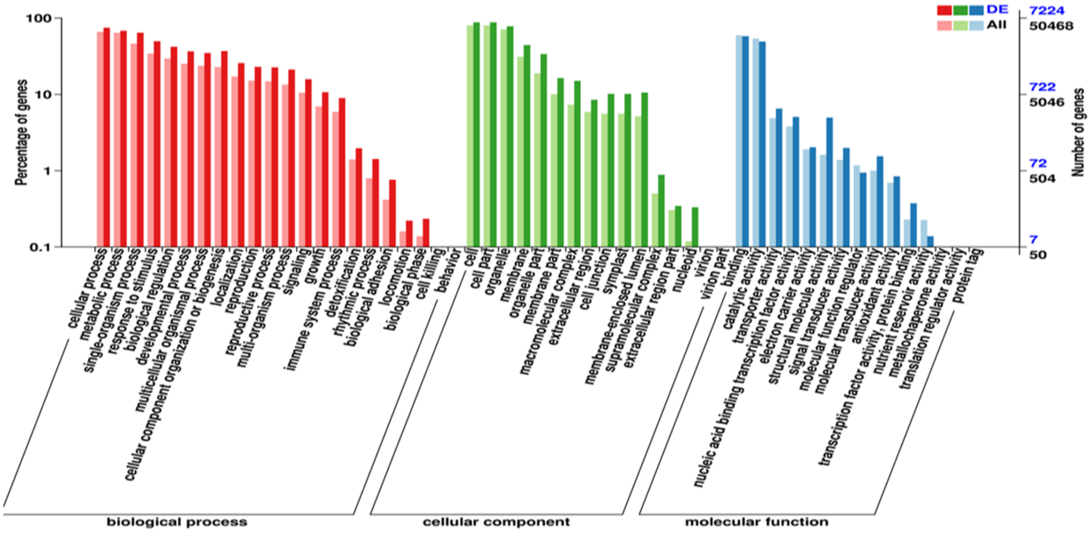

Supplementary file 1: Figure S4. GO analysis of differentially expressed genes classification into three main categories, biological process, molecular function and cellular component. The y-axis indicates the number of DEGs in a category. In the three main GO categories in H1 compared with *ny2*, there were 22, 16, and 15 functional groups, respectively

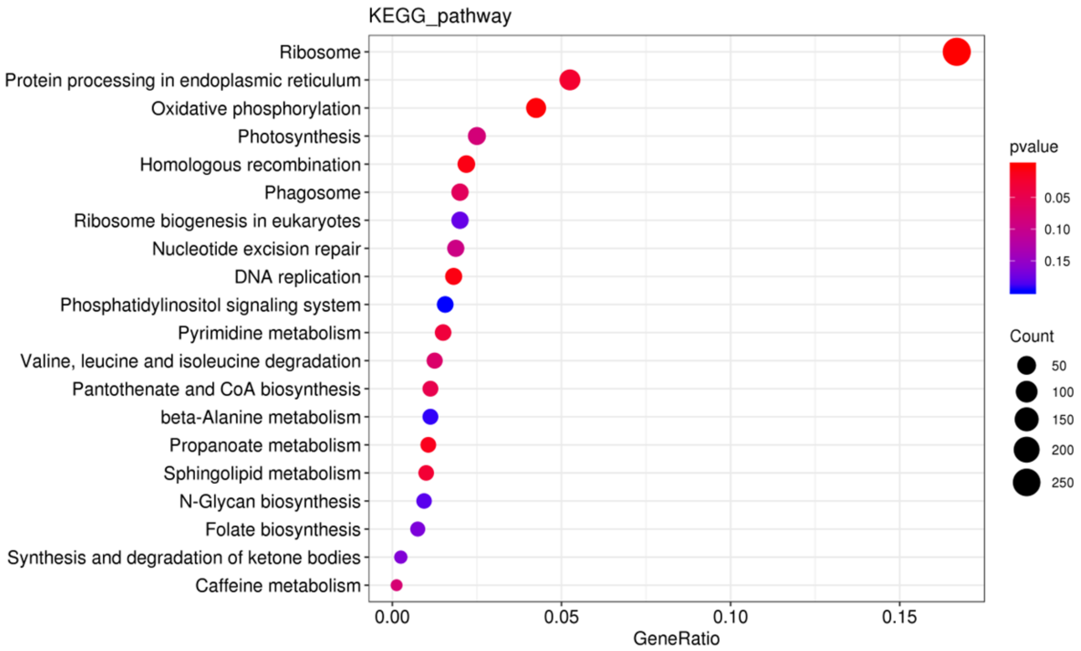

Supplementary file 1: Figure S5. KEGG pathways enriched between *ny2* and H1 (WT) during meiosis

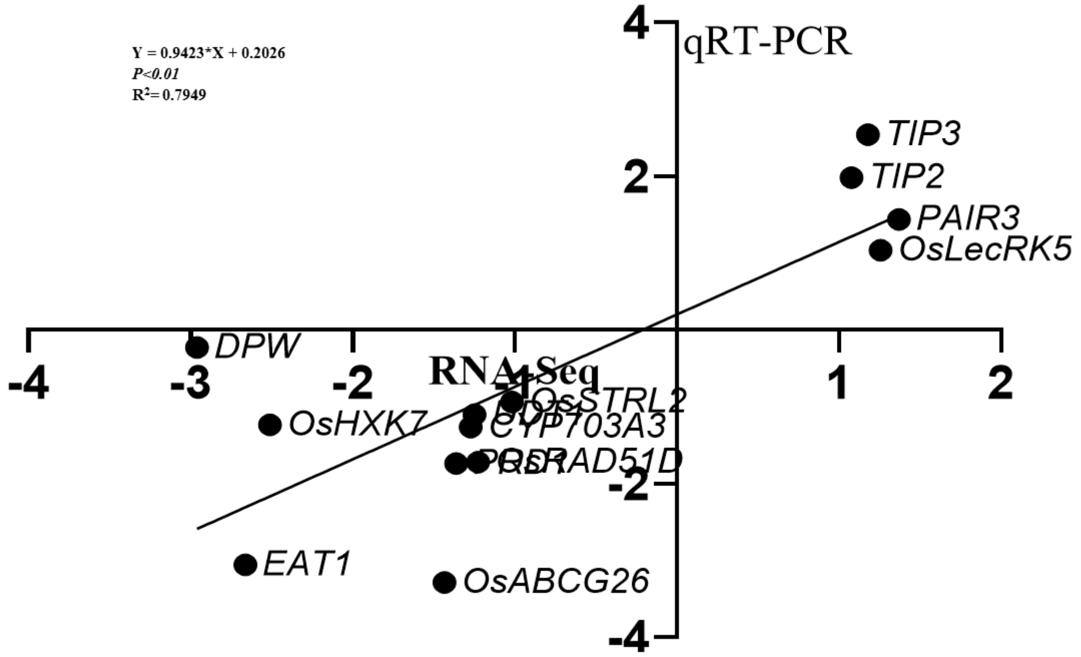

Supplementary file 1: Figure S6 Comparison of the log2 (FC) of 13 selected genes using qRT-PCR analysis in WT compared with *ny2*.

Note: Both the $\log_2$ transformed values of relative expression of qRT-PCR and fold changes of RNA seq data were used for linear regression. The Pearson's correlation coefficient was used to measure the linear correlation of qRT-PCR and RNA seq analysis
